# Dual Sites for SEC11 on the SNARE SYP121 Implicate a Binding Exchange during Secretory Traffic^1^

**DOI:** 10.1104/pp.18.01315

**Published:** 2019-03-08

**Authors:** Ben Zhang, Rucha Karnik, Jonas Alvim, Naomi Donald, Michael R. Blatt

**Affiliations:** a School of Life Science, Shanxi University, Taiyuan 030006, China; b Laboratory of Plant Physiology and Biophysics, University of Glasgow, Glasgow G12 8QQ, United Kingdom

## Abstract

SNARE (soluble *N*-ethylmaleimide-sensitive factor attachment protein receptor) proteins facilitate vesicle traffic through their assembly in a heteromeric complex that drives membrane fusion. Much of vesicle traffic at the Arabidopsis (*Arabidopsis thaliana*) plasma membrane is subject to the Sec1/Munc18 protein SEC11, which, along with plasma membrane K^+^ channels, selectively binds with the SNARE SYP121 to regulate its assembly in complex. How SEC11 binding is coordinated with the K^+^ channels is poorly understood, as both SEC11 and the channels are thought to compete for the same SNARE binding site. Here, we identify a second binding motif within the N terminus of SYP121 and demonstrate that this motif affects SEC11 binding independently of the F^9^xRF motif that is shared with the K^+^ channels. This second, previously unrecognized motif is centered on residues R^20^R^21^ of SYP121 and is essential for SEC11 interaction with SYP121. Mutation of the R^20^R^21^ motif blocked vesicle traffic without uncoupling the effects of SYP121 on solute and K^+^ uptake associated with the F^9^xRF motif; the mutation also mimicked the effects on traffic block observed on coexpression of the dominant-negative SEC11^Δ149^ fragment. We conclude that the R^20^R^21^ motif represents a secondary site of interaction for the Sec1/Munc18 protein during the transition of SYP121 from the occluded to the open conformation that leads to SNARE complex assembly.

## Abstract (precis)

The regulatory protein SEC11 binds differentially via two motifs on the Qa-SNARE SYP121 N terminus, only one of which overlaps with the K^+^ channel-binding motif, to facilitate a binding exchange during SNARE complex assembly.

SNAREs (soluble *N*-ethylmaleimide-sensitive factor attachment protein receptors) comprise a highly conserved superfamily of proteins in all eukaryotic cells and play important roles in membrane fusion for targeting and delivery of membranes, proteins, and soluble cargos. They form a core complex to bring vesicle and target membrane surfaces together, thereby driving secretion as well as the traffic of vesicles between endosomal compartments. SNAREs are classified as either target- or vesicle-SNAREs, reflecting their functional localization, and as either Q (Gln)- or R (Arg)-SNAREs, based on the conserved amino acid contributing to the central layer of the core complex. The Q-SNAREs are further divided between Qa-, Qb-, and Qc-SNARE forms, one of each contributing to the core complex with the cognate R-SNARE. In plant cells, SNAREs are important for biotic and abiotic stress resistance, homeostasis, cell expansion, and growth (Blatt, 2000; Shope et al., 2003; Lipka et al., 2007; Bassham and Blatt, 2008; Karnik et al., 2017). A unifying theme of SNARE action in these processes is in SNARE complex assembly and vesicle traffic.

Beyond the canonical role in membrane fusion, a few SNAREs are also known to interact with ion channels and affect their regulation. The plasma membrane Qa-SNARE SYP121 (=SYR1/PEN1) of Arabidopsis (*Arabidopsis thaliana*) is the best-known example. SYP121, and its close homolog SYP122, have similar structures and overlapping functions in facilitating secretory traffic in most tissues in the plant (Bassham and Blatt, 2008; Waghmare et al., 2018). They are expressed throughout vegetative growth (Uemura et al., 2004; Enami et al., 2009) and, during SNARE complex formation, share the partners SNAP33, a Qbc-SNARE, and VAMP721 and VAMP722, two nearly identical R-SNAREs (Kwon et al., 2008; Karnik et al., 2013b, 2015). However, SYP121 also interacts with the K^+^ channels KC1 and KAT1, altering channel gating to promote K^+^ uptake (Honsbein et al., 2009). Channel binding is specific for SYP121: it depends on a conserved N-terminal motif defined by the sequence F^9^xRF within SYP121, and this motif is missing from SYP122 (Grefen et al., 2010a). VAMP721 also binds the KC1 and KAT1 K^+^ channels, but its action is to suppress channel gating in a manner opposing that of SYP121 (Zhang et al., 2015). These and additional results suggest a temporal exchange of the K^+^ channels between the cognate SNAREs SYP121 and VAMP721 to regulate channel activity while concurrently promoting vesicle traffic. Indeed, SYP121-K^+^ channel interactions are known to confer a voltage dependence on secretory traffic for growth, supporting the idea of vesicle traffic that is accelerated with osmotic solute uptake through these channel interactions (Grefen et al., 2015; Karnik et al., 2017).

A critical and yet unanswered question is how such vesicle traffic is mediated in conjunction with other interacting factors, notably with SEC11 (=KEULE). SEC11 is a member of the Sec1/Munc18 (SM) protein family that is now recognized to regulate SNARE complex assembly (Misura et al., 2000; Südhof and Rothman, 2009; Archbold et al., 2014). SM proteins fold into an arch-shaped clasp with a major cleft that binds secretory Qa-SNAREs in animals and yeast to prevent their promiscuous interaction with other SNARE proteins, and they also bind with the SNARE complex, once assembled, to stabilize the complex and accelerate vesicle fusion (Südhof and Rothman, 2009; Hughson, 2013). SEC11 is expressed throughout the vegetative plant, although it was originally described to bind with SYP111 (=KNOLLE), which is expressed only during cytokinesis (Waizenegger et al., 2000; Assaad et al., 2001). Karnik et al. (2013b) have shown that SEC11 binds SYP121 and plays a role in traffic at the plasma membrane of nondividing cells. Like other SM proteins, SEC11 incorporates a minor cleft that interacts with the N terminus of SYP121 and is important for the conformational changes that lead to SNARE complex assembly (Karnik et al., 2013b, 2015). Binding of the SEC11 minor cleft depends on the F^9^ residue within the F^9^xRF motif of the SNARE. Thus, it has been suggested that SM and channel proteins may compete for SYP121 (Grefen et al., 2010a).

SEC11 binding distinguishes secretory traffic mediated by SYP121 from that mediated by SYP122. Notably, SEC11 binds preferentially with SYP121 and rescues traffic block by dominant-negative fragments of the SNAREs when these fragments are expressed, but only in plants that also express the native SYP121 (Karnik et al., 2015). Furthermore, the same requirement for the native SYP121 applies to block by the SEC11^Δ149^ fragment, which includes the SM minor cleft and retains Qa-SNARE binding in vitro and in vivo (Karnik et al., 2015). The protein sequences of SYP121 and SYP122 exhibit a substantial degree of identity (Grefen et al., 2010a), including F^9^ within the SNARE N terminus. Thus, it is likely that the common F^9^ and adjacent residues are not sufficient to determine the SEC11-SNARE specificity or the joint interactions of SYP121 with both SEC11 and the K^+^ channels. Identifying the additional motif(s) of SYP121 for binding with the SEC11 minor cleft will likely clarify the mechanics of SEC11 and the K^+^ channels within the SNARE cycle of SYP121.

Here, we identify a second, N-terminal SEC11-binding motif associated with residues R^20^R^21^ of SYP121. We show that this motif is essential but distinguishable in its SNARE-binding characteristics from the F^9^xRF motif of SYP121. Moreover, we demonstrate that the R^20^R^21^ motif is important for the selectivity of SEC11 for SYP121 in secretory traffic and for the link between vesicle traffic and osmotic solute uptake during plant growth.

## RESULTS

### SEC11 Interacts Preferentially with SYP121

**Figure 1.**
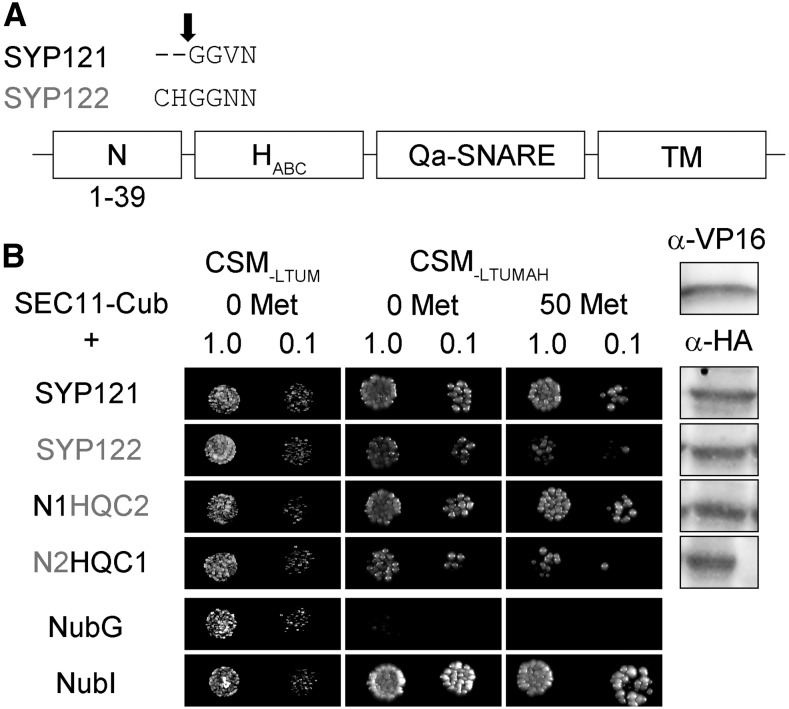
SEC11 interaction with SYP121 depends on the presence of the N terminus of the Qa-SNARE. A, Segment alignments (top) for SYP121 and SYP122 at the junction point, with the arrow indicating the domain breaks. B, Diploid yeast expressing SEC11-Cub as bait with NubG-X fusions of SNARE proteins and controls (negative, NubG; positive, NubI) as prey were spotted onto different media as indicated. Growth on CSM_-LTUM_ was used to verify the presence of both bait and prey expression. CSM_-LTUMAH_ was used to verify Ade- and His-independent growth of the yeast diploids. The addition of 50 μm Met to CSM_-LTUMAH_ was used to verify interaction with SEC11-Cub expression suppressed. Yeast was dropped at 1 and 0.1 OD_600_ as indicated. Incubation time was 24 h for CSM_-LTUM_ plate and 72 h for CSM_-LTUMAH_ plates. Immunoblot analysis (5 μg of total protein per lane) of the haploid yeast used in mating (right) used the α-HA antibody for the SNARE fusions and the α-VP16 antibody for the SEC11 fusion.

We made use of a mating-based split-ubiquitin (mbSUS) assay for protein-protein interaction analysis (Grefen et al., 2007), previously employed successfully to identify SNARE-K^+^ channel (Honsbein et al., 2009; Grefen et al., 2010a, 2015; Zhang et al., 2015) and SNARE-SEC11 (Karnik et al., 2015) interactions. In the mbSUS assay, interactions between proteins fused with the N- and C-terminal halves (Nub and Cub) of the ubiquitin moiety facilitate their reassembly, leading to transactivator cleavage, reporter gene activation, and yeast growth on selective media. This assay incorporates a number of advantages over similar protein interaction screens, including bait suppression in the presence of Met as a test for the specificity of interaction. As shown in Figure 1, diploid yeast expressing SEC11-Cub as bait with SYP121 grew well on selective media, even in the presence of 50 μm Met. Growth was weaker with SYP122 as the prey, consistent with the preferential binding and action of SEC11 with SYP121 (Karnik et al., 2013b, 2015).

**Figure 2.**
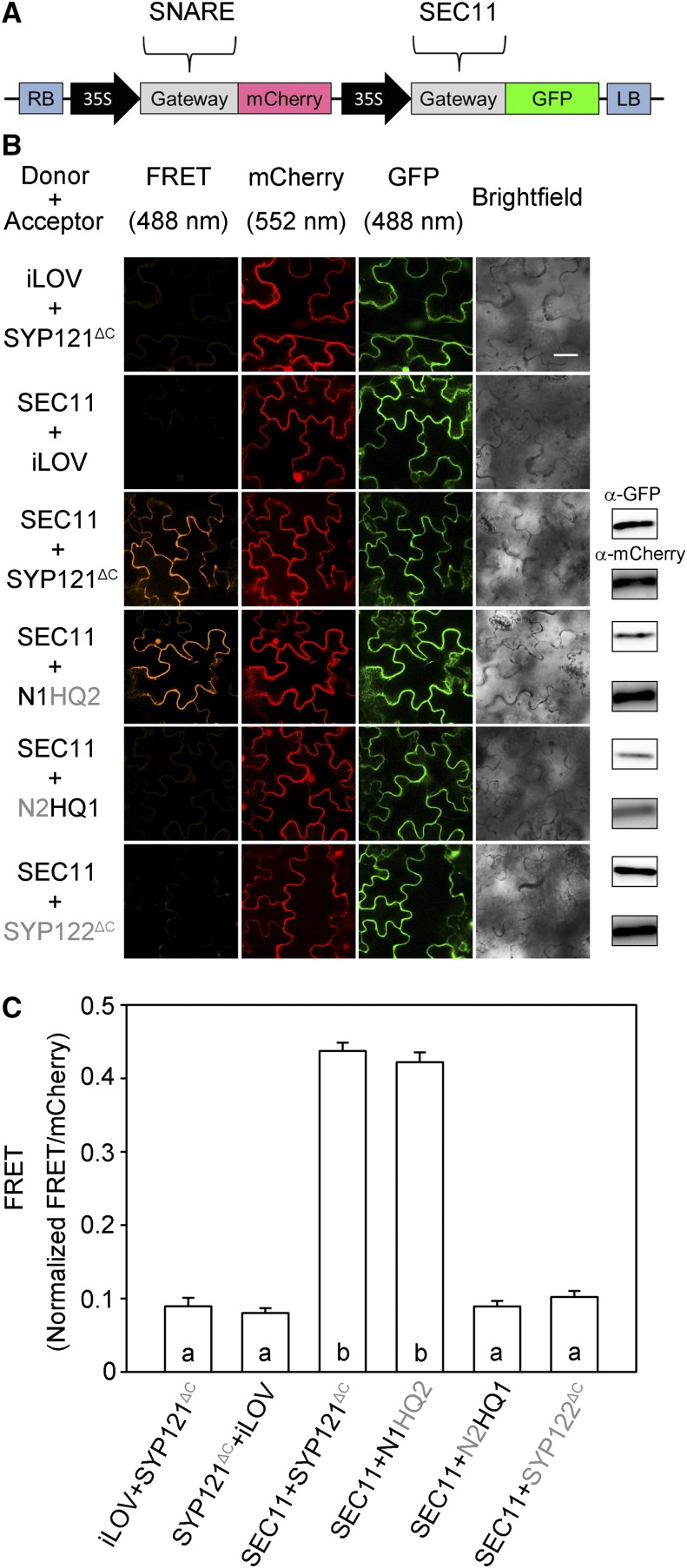
SEC11 interaction with SYP121 depends on the presence of the N-terminal domain of the SNARE in vivo. FRET analysis was performed for SEC11 interaction with SYP121^ΔC^, SYP122^ΔC^, and the SYP121 chimeras. The FRET, mCherry, and GFP fluorescence signals were collected from tobacco (*Nicotiana tabacum*) leaf epidermis transformed using the 2in1 vector (Hecker et al., 2015). A, Schematic of the 2in1 vectors used for FRET analysis (Hecker et al., 2015). The SEC11-GFP fusion was used as donor, and SNARE-mCherry fusion was used as acceptor. LB, Left border; RB, right border. B, Images at uniform magnification are (left to right) mCherry (FRET) fluorescence (excitation, 488 nm), mCherry fluorescence (excitation, 552 nm), GFP fluorescence (excitation, 488 nm), and bright field. Constructs (top to bottom) expressed iLOV-GFP with SYP121^ΔC^-mCherry, SEC11-GFP with iLOV-mCherry as negative control, and SEC11-GFP with SYP121^ΔC^, SYP122 ^ΔC^, and chimeras. Immunoblot analysis is shown at right with anti-GFP (top) and anti-mCherry (below) antibodies to verify fusion protein expression. Bar = 20 μm. C, FRET fluorescence signals from three independent experiments. Each bar represents the mean ± se of fluorescence intensity ratios from 10 images per experiment, taken at random over the root surface. Background fluorescence was subtracted as determined from nontransformed roots. FRET signals were calculated as the mean fluorescence intensity ratio between FRET and mCherry after correcting for bleedthrough and normalizing to the GFP signal. Significant differences (*P* < 0.05) are indicated by different letters.

**Figure 3.**
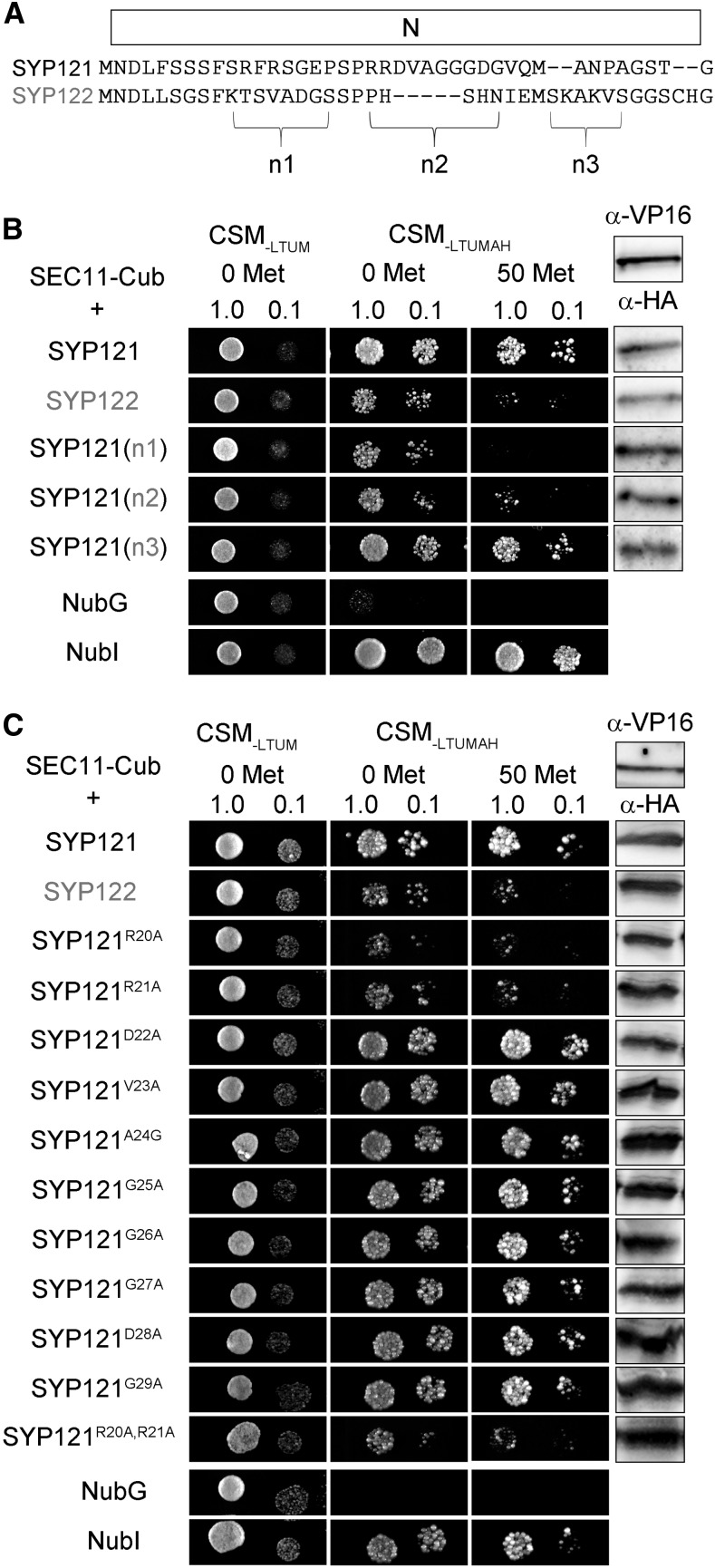
Interaction with SEC11 depends on R^20^R^21^ of SYP121. A, Alignment of the segments for SYP121 and SYP122 within the N-terminal domain. The n1, n2, and n3 segments are indicated. B, Diploid yeast expressing SEC11-Cub as bait with NubG-X fusions of SNARE chimeras and controls (negative, NubG; positive, NubI) as prey were spotted onto different media as indicated. N-terminal chimeras were constructed by replacement of the n1, n2, and n3 segments from SYP122 in SYP121. Growth on CSM_-LTUM_ was used to verify the presence of both bait and prey expression. CSM_-LTUMAH_ was used to verify Ade- and His-independent growth of the yeast diploids. The addition of 50 μm Met to CSM_-LTUMAH_ was used to verify interaction with SEC11-Cub expression suppressed. Yeast was dropped at 1 and 0.1 OD_600_ as indicated. Incubation time was 24 h for CSM_-LTUM_ plate and 72 h for CSM_-LTUMAH_ plates. Immunoblot analysis (5 μg of total protein per lane) of the haploid yeast used in mating (right) used the α-HA antibody for the SNARE fusions and the α-VP16 antibody for the SEC11 fusion. C, Diploid yeast expressing SEC11-Cub as bait with NubG-X fusions of SYP121 carrying substitutions with Ala at the n2 segment and controls (negative, NubG; positive, NubI) as prey were spotted onto different media as indicated. Growth on CSM_-LTUM_ was used to verify the presence of both bait and prey expression. CSM_-LTUMAH_ was used to verify Ade- and His-independent growth of the yeast diploids. The addition of 50 μm Met to CSM_-LTUMAH_ was used to verify interaction with SEC11-Cub expression suppressed. Yeast was dropped at 1 and 0.1 OD_600_ as indicated. Incubation time was 24 h for CSM_-LTUM_ plate and 72 h for CSM_-LTUMAH_ plates. Immunoblot analysis (5 μg of total protein per lane) of the haploid yeast used in mating (right) used the α-HA antibody for the SNARE fusions and the α-VP16 antibody for the SEC11 fusion.

To verify this selectivity in SEC11 interaction between the two SNAREs in vivo, we used Förster resonance energy transfer (FRET) analysis (Hecker et al., 2015) after substituting the C-terminal membrane anchor domains of SYP121 and SYP122 with mCherry as the fluorescence energy acceptor and with SEC11-GFP as the energy donor. Previous studies showed that the soluble domains of the SNAREs retain the ability to interact with SEC11 (Karnik et al., 2013b). We used the 2in1 vector system that incorporates a set of independent Gateway-compatible cassettes, enabling cotransformations with a single vector for equal genetic loads on transformation (Fig. 2A; Hecker et al., 2015). The soluble iLOV protein was also cloned into this system as a negative control. iLOV comprises the kinase and LOV-Jα domains of the PHOT1 photoreceptor protein (Chapman et al., 2008) and is unrelated to the SNAREs. Figure 2B shows representative FRET images with supporting immunoblots from one experiment with each of the constructs, and Figure 3C summarizes the data from three independent experiments. Additionally, we used the mCherry fluorescence excited at 552 nm as a reference for comparisons. mCherry fluorescence excited by 488-nm light showed strong and highly significant signals when SEC11 was coexpressed with the SYP121 construct and with chimeras incorporating the N-terminal domain of SYP121, but not with SYP122 chimeras with the N-terminal domain of SYP122 or with the iLOV control. These results verified that the selectivity of SEC11 binding with SYP121 depends on its N terminus.

### A Second, N-Terminal Motif for SYP121 Binding with SEC11

An alignment of the N termini of SYP121 and SYP122 shows that the SNAREs differ principally across three short segments (designated n1, n2, and n3 in Fig. 3A). We used these sequences from SYP122, substituting them into the corresponding positions of SYP121 for interaction analysis with the SEC11-Cub bait (Fig. 3B). SEC11 interaction was suppressed in the constructs that incorporated the n1 and n2 segments from SYP122. The n1 segment spans the critical F^9^xRF motif, previously identified also with K^+^ channel binding (Grefen et al., 2010a). Sensitivity to substitution of the n2 segment suggested a second motif for SEC11 binding located some 10 residues or more away from the F^9^xRF motif. To identify this second motif, we used Ala substitution mutagenesis, targeting each of the 10 residues from the n2 segment of SYP121 for analysis by mbSUS assay with SEC11-Cub as bait. Figure 3C shows that yeast growth was strong in every case except when the mutations SYP121^R20A^ and SYP121^R21A^ were used as prey. Growth was suppressed, especially in the presence of 50 μm Met to reduce bait expression, and similar results were observed with the double mutant SYP121^R20AR21A^, indicating that these residues are important for SEC11 interaction.

**Figure 4.**
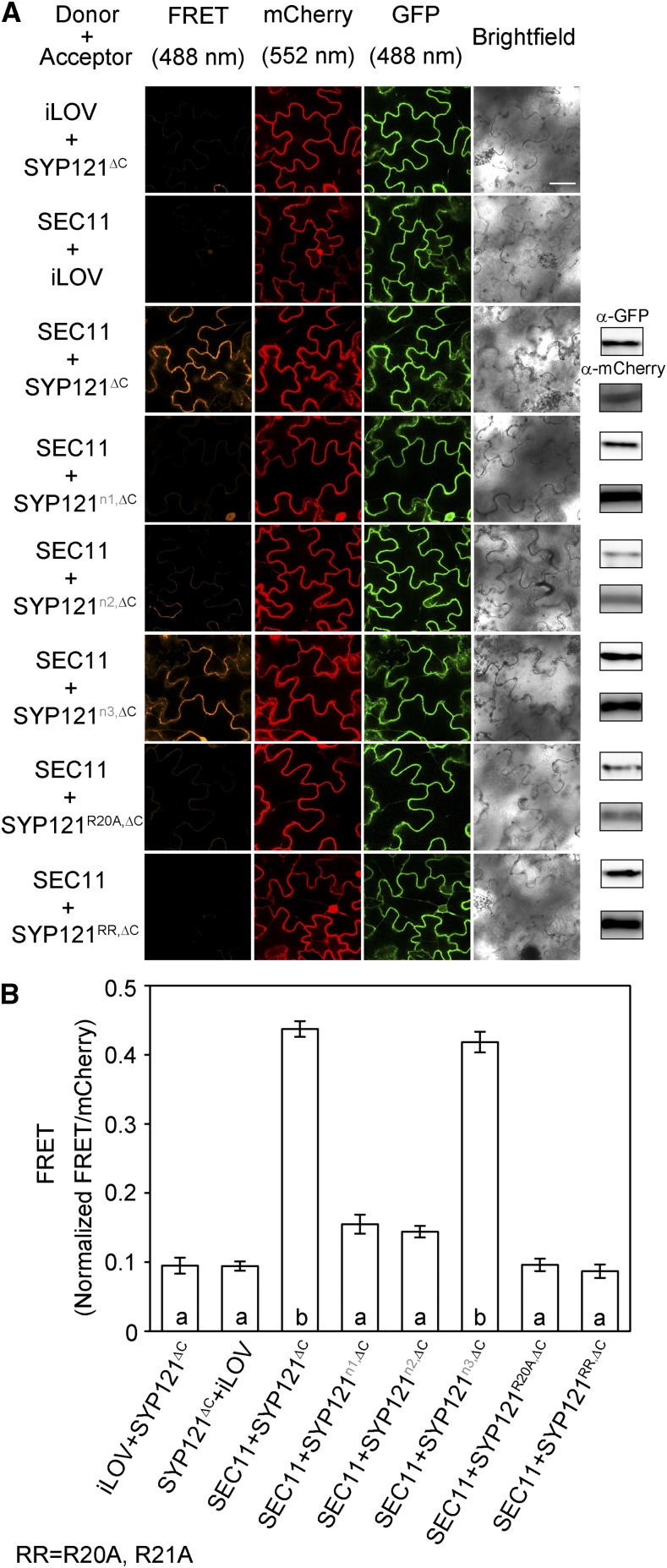
Interaction in vivo with SEC11 depends on the R^20^R^21^ motif of SYP121. FRET analysis was performed for SEC11 interaction with SYP121^ΔC^, SYP122^ΔC^, and chimeras. The FRET, mCherry, and GFP fluorescence signals were collected from tobacco leaf epidermis transformed using the 2in1 vector (Hecker et al., 2015). A, Images at uniform magnification are (left to right) mCherry (FRET) fluorescence (excitation, 488 nm), mCherry fluorescence (excitation, 552 nm), GFP fluorescence (excitation, 488 nm), and bright field. From top to bottom, Arabidopsis seedlings expressed iLOV-GFP with SYP121^ΔC^-mCherry, SEC11-GFP with iLOV-mCherry as the negative control, SEC11-GFP with SYP121^ΔC^-mCherry as the positive control, and constructs expressing SEC11-GFP with segment chimeras and the Ala-substituted R^20^R^21^ double mutant of SYP121^ΔC^-mCherry. Immunoblot analysis is shown at right with anti-GFP (top) and anti-mCherry (below) antibodies to verify fusion protein expression. Bar = 20 μm. B, FRET fluorescence signals from three independent experiments. Each bar represents the mean ± se of fluorescence intensity ratios of 10 images per experiment, taken at random over the root surface, after subtracting the background fluorescence determined from nontransformed roots. FRET signals were calculated as the mean fluorescence intensity ratio between FRET and mCherry after correcting for bleedthrough and normalizing to the GFP signal. Significant differences (*P* < 0.05) are indicated by different letters.

To validate these findings in vivo, we performed the FRET analysis using the corresponding n1, n2, and n3 segment chimeras as well as the site mutants. Figure 4 shows representative images and statistical analyses of FRET ratios from three independent experiments. As with SEC11, we recovered FRET signals with the wild-type and n3 segment substitutions. However, an appreciable FRET signal was absent when the SEC11-GFP donor was expressed with SYP121 chimeras containing n1 or n2 segment substitutions from SYP122, with the SYP121^R20A^ mutant, and with the SYP121^R20AR21A^ double mutant. Separate experiments confirmed that the SYP121^R20AR21A^ double mutant, like the wild-type Qa-SNARE, localized to the cell periphery (Supplemental Fig. S1). These results demonstrate a requirement for residues R^20^ and R^21^ in SYP121 to interact with SEC11. Thus, we conclude that there are two distinct SEC11-binding motifs within the SYP121 N terminus.

### SYP121^R20A,R21A^-Associated Mutations Affect SEC11^Δ149^ Peptide Binding, Secretory Traffic, and Growth

**Figure 5.**
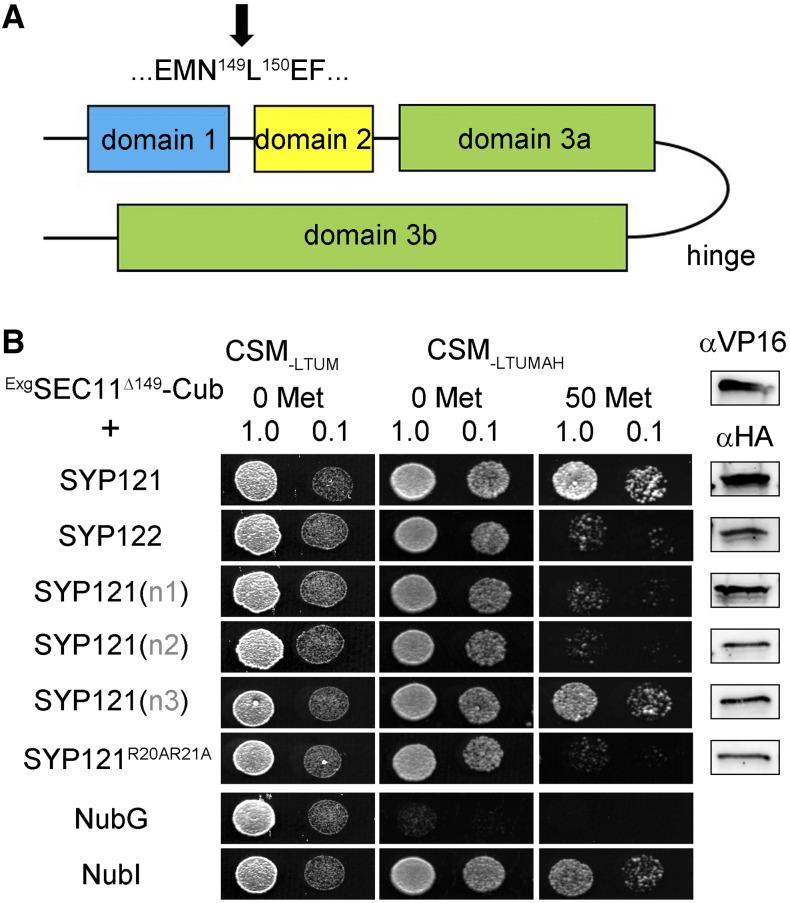
The SYP121 R^20^R^21^ motif affects binding with SEC11^Δ149^. A, Schematic of SEC11 domain topology. The domain break is indicated by the arrow. B, Yeast mbSUS assay is performed by using Exg2p fusions of SEC11-Cub (Zhang et al., 2018) as bait and NubG-X fusions of SNARE proteins and controls (negative, NubG; positive, NubI) as prey. Growth on CSM_-LTUM_ was used to verify the presence of both bait and prey expression. CSM_-LTUMAH_ was used to verify Ade- and His-independent growth of the yeast diploids. The addition of 50 μm Met to CSM_-LTUMAH_ was used to verify interaction with SEC11-Cub expression suppressed. Yeast was dropped at 1 and 0.1 OD_600_ as indicated. Incubation time was 24 h for CSM_-LTUM_ plate and 72 h for CSM_-LTUMAH_ plates. Immunoblot analysis (5 μg of total protein per lane) of the haploid yeast used in mating (right) used the α-HA antibody for the SNARE fusions and the α-VP16 antibody for the SEC11 fusion.

SEC11, like other SM family proteins, consists of four structural elements (Karnik et al., 2015) that assemble three domains, two in the N-terminal half and a hinge that divides the third and largest domain to form a clamp-like structure (Fig. 5A). Domain 1 of yeast and mammalian SM proteins binds to the N terminus of the cognate Qa-SNAREs and is thought to anchor the SM protein while facilitating Qa-SNARE conformational transitions to assemble with its cognate SNARE partners (Furgason et al., 2009; Südhof and Rothman, 2009). The same domain of SEC11 is sufficient to bind with SYP121 in Arabidopsis: Karnik et al. (2015) found that expressing this first 149-amino acid sequence of SEC11, which incorporates the minor cleft of the SM protein, was able to bind SYP121 and block secretory traffic.

To assess whether the R^20^R^21^ motif in SYP121 affects binding of SEC11 through this N-terminal domain, we performed the SUS assay in yeast using the SEC11^Δ149^ peptide as bait. To anchor the peptide to the membrane, we fused SEC11^Δ149^ with a glycosyl-phosphatidylinositol signal peptide derived from glucan 1,3-β-glucosidase2 (Exg2), a fusion that successfully recapitulates previous pulldown studies with the SEC11^Δ149^ peptide and the cytosolic domain of SYP121 (Karnik et al., 2013b, 2015; Zhang et al., 2018). As shown in Figure 5B, diploid yeast expressing the Exg2-SEC11^Δ149^ bait protein showed strong growth with the SYP121 prey constructs as before, provided that the prey included both the n1 and n2 domains of SYP121 but not when either of these domains was substituted with the corresponding domains of SYP122 or when the SYP121^R20A,R21A^ mutant was used as prey. These results indicate that the R^20^R^21^ motif is also essential for binding of SEC11^Δ149^ with SYP121.

**Figure 6.**
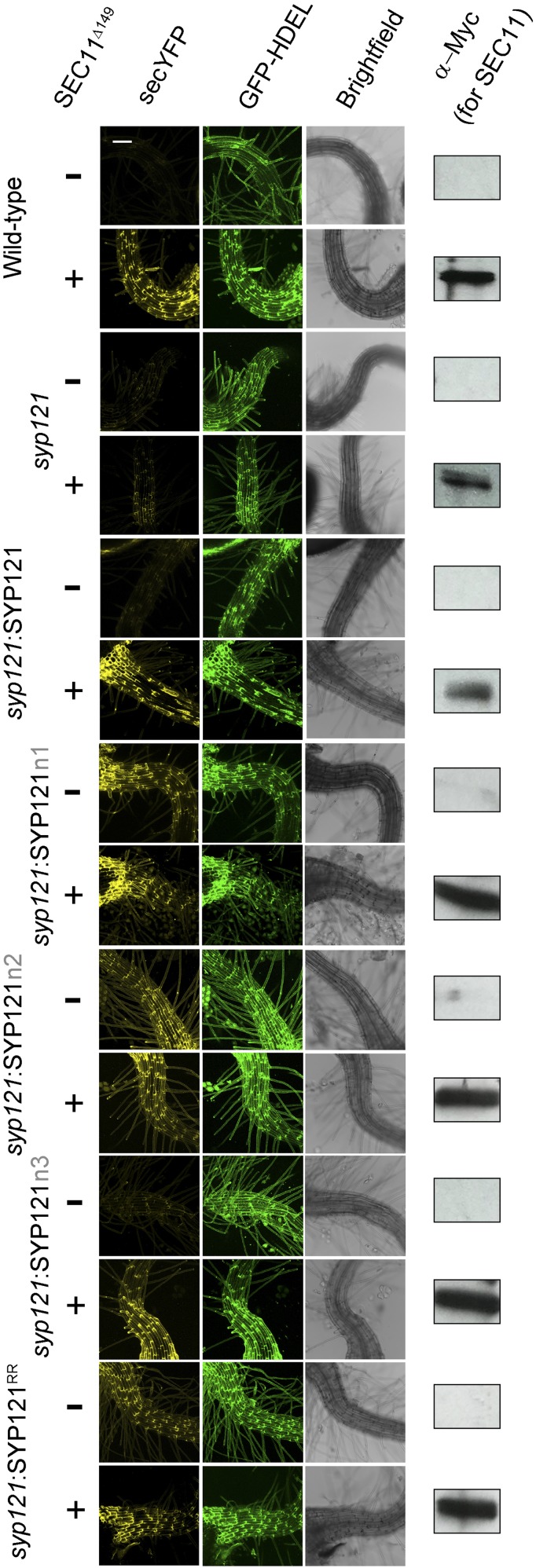
*syp121* complementation with SYP121^R20AR21A^ alone blocks secretory traffic independent of SEC11^Δ149^. Images at uniform magnification are (left to right) 3D projections of fluorescence signals with the secretory marker secreted yellow fluorescent protein (secYFP), the retained (endoplasmic reticulum) marker GFP-HDEL, and bright field. The Arabidopsis *syp121* mutant was complemented with SYP121, SYP121n1, SYP121n2, SYP121n3, and SYP121^R20AR21A^ driven by the UBQ10 promoter (Grefen et al., 2010b). The wild type and the *syp121* line were included as controls. The secYFP and the endoplasmic reticulum marker GFP-HDEL were expressed with or without SEC11^Δ149^ fragment by using the pTecG-2in1-CC vector (Karnik et al., 2013b, 2015). Immunoblot analysis verifying the expression of SEC11^Δ149^ with the anti-myc antibody is shown at right. Bar = 100 μm.

**Figure 7.**
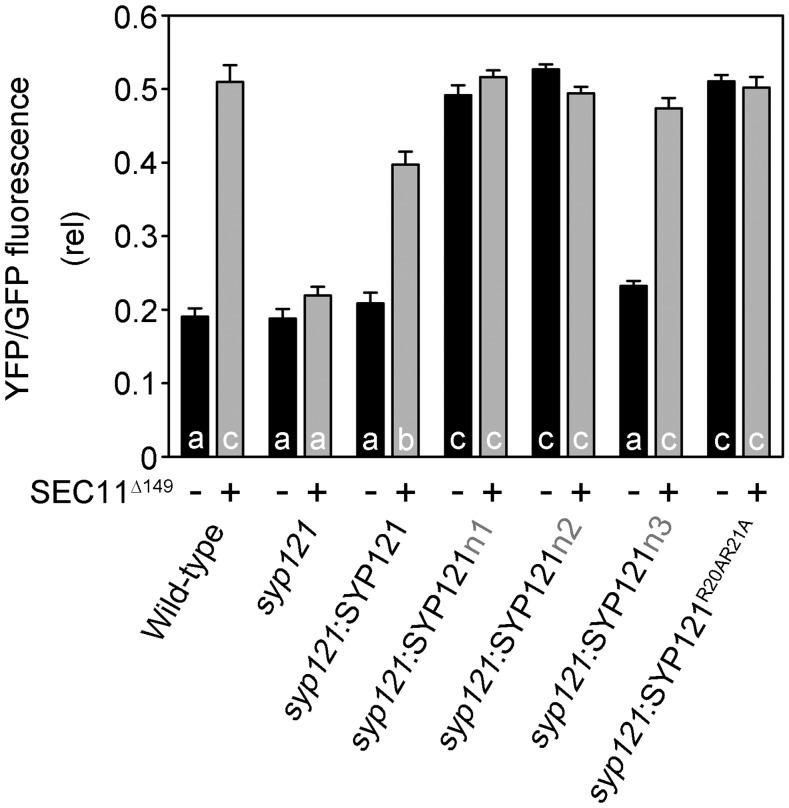
Analysis of SYP121^R20AR21A^ secretory block. Secretory traffic was calculated as YFP-GFP fluorescence ratios from three independent experiments (high YFP-GFP ratio indicates secretory block). Each bar represents the mean ± se of fluorescence intensity ratios of 10 images per experiment, taken at random over the root surface. Significant differences (*P* < 0.05) are indicated by different letters.

Previous studies had indicated that overexpressing the SEC11^Δ149^ peptide blocks secretory traffic in wild-type Arabidopsis but not in the *syp121* mutant (Karnik et al., 2015). To investigate the effect on secretory traffic of the two N-terminal motifs of SEC11 and its binding to SYP121, we generated stable lines of Arabidopsis in the *syp121* mutant background expressing SYP121, SYP121n1, SYP121n2, SYP121n3, and SYP121^R20A,R21A^ driven by the moderate Ubiquitin10 (UBQ10) promoter (Grefen et al., 2010b) and verified the expression of the transgenes by immunoblot analysis (Supplemental Fig. S2). Secreted YFP and GFP-HDEL were expressed in these lines after transient transformation using the pTecG-2in1-CC vector both without and with a cassette encoding the SEC11^Δ149^ peptide, much as before (Karnik et al., 2013b, 2015). Figure 6 shows one set of fluorescence images and Figure 7 summarizes the data analysis from three independent experiments using the YFP-GFP ratio to quantify secretion. Overexpressing the SEC11^Δ149^ peptide led to a significant retention of secYFP in wild-type plants and *syp121* mutant plants complemented with SYP121 but not in the *syp121* mutant. These findings are consistent with previous results and validated the assay (Karnik et al., 2015). In the *syp121* mutant background, expressing SYP121n3 yielded results similar to those of the wild type: coexpressing the SEC11^Δ149^ peptide blocked secYFP traffic, as evidenced by the increase in the YFP-GFP ratio. However, expressing SYP121n1, SYP121n2, or SYP121^R20AR21A^ alone was sufficient to suppress secYFP secretion. Furthermore, cotransformations to express the SEC11^Δ149^ peptide in these backgrounds had no additional effect on secYFP traffic. We interpret these findings in the context of a loss of binding with the SEC11^Δ149^ peptide. That expressing the SYP121 chimeras and SYP121^R20AR21A^ alone led to secYFP retention is consistent with traffic block associated with a loss of SEC11 anchoring to the SYP121 N terminus (Karnik et al., 2013b, 2015), and we return to these points in “Discussion.”

**Figure 8.**
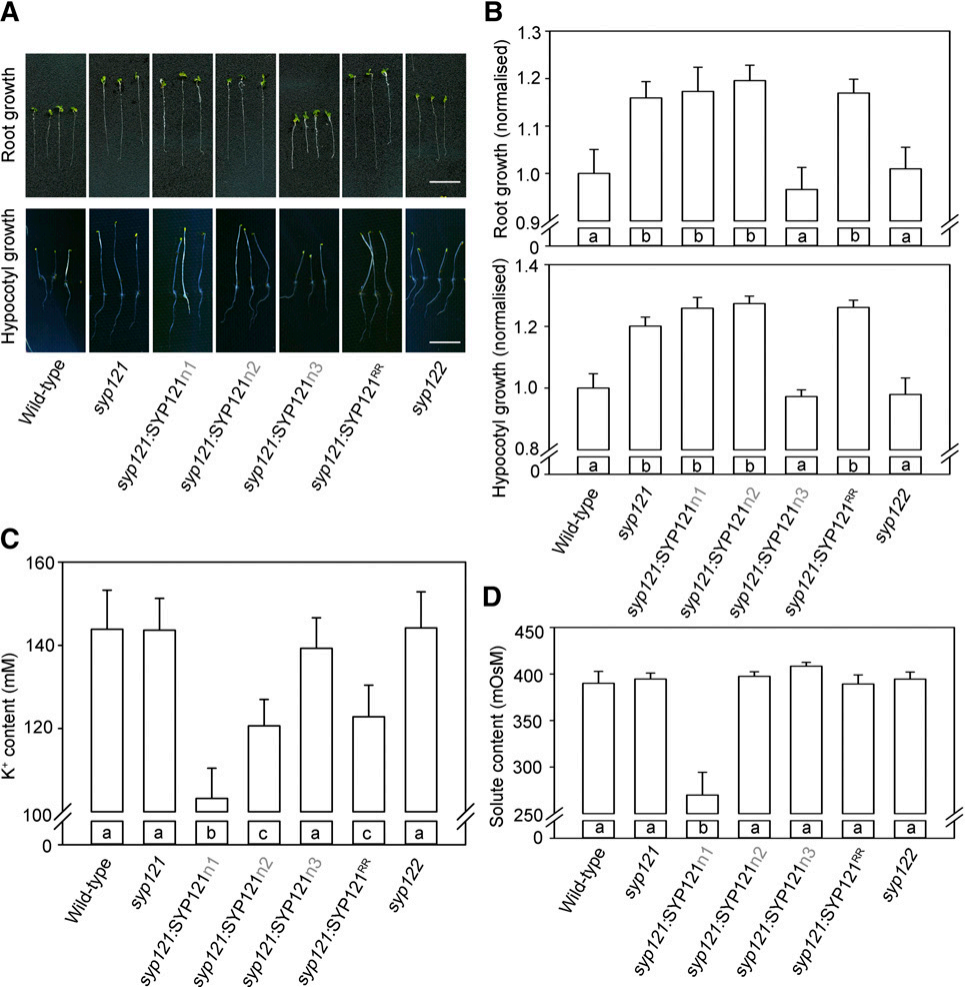
*syp121* complementation with SYP121^R20AR21A^ affects Arabidopsis growth but only to a lesser extent affects the K^+^ and osmotic contents. A, Root (top) and hypocotyl (bottom) growth of *syp121* Arabidopsis lines stably expressing SYP121, SYP121n1, SYP121n2, SYP121n3, and SYP121^R20AR21A^ under the control of the UBQ10 promoter (Grefen et al., 2010b); the wild type and *syp121* and *syp122* lines were used as controls. For root measurements, plants were grown for 10 d in liquid culture with defined minimal salts medium including 1 mm K^+^ at 22°C and 200 μmol m^−2^ s^−1^ photosynthetically active radiation (Honsbein et al., 2009). For hypocotyl growth measurements, seed germination was activated by placing the plates under a red filter for 3 h and then transferred to darkness for 6 d. Bars = 1 cm. B, Root (top) and hypocotyl (bottom) lengths of Arabidopsis lines depicted in A determined as means ± se from three independent experiments with more than 10 seedlings for each line. All lengths are normalized to that of the wild type grown in parallel. Significant differences (*P* < 0.05) are indicated by different letters. C, Mean ± se of total K^+^ contents of seedlings collected from the same plants used for root growth measurements. Significant differences (*P* < 0.05) are indicated by different letters. D, Mean ± se of total osmotic contents as in C. Significant differences (*P* < 0.05) are indicated by different letters.

Finally, to determine whether SEC11 binding associated with the SYP121 R^20^R^21^ motif affected plant growth, we examined the same stable lines expressing SYP121n1, SYP121n2, SYP121n3, and SYP121^R20AR21A^ in the *syp121* background. Plants derived from at least two independent lines for each construct were analyzed for root length, hypocotyl length, and osmotic and K^+^ contents. Wild-type Arabidopsis and the *syp121* and *syp122* mutant lines were included as controls. As shown in Figure 8, root and hypocotyl growth and the osmotic and K^+^ contents of *syp122* lines were similar to those of wild-type plants. However, growth was promoted in the *syp121* lines, and similar results were obtained in lines expressing SYP121n1, SYP121n2, and SYP121^R20AR21A^ in the *syp121* mutant background. Significantly, both K^+^ and osmotic contents were reduced in the lines expressing SYP121n1, consistent with the loss of K^+^ channel binding. In the lines expressing SYP121n2 and SYP121^R20AR21A^, the K^+^ content showed a decrease that was intermediate between that of the wild type and the SYP121n1 mutant. These results show a clear distinction between binding associated with the n1 domain and the F^9^xRF motif, which is important for interaction with both the K^+^ channels and SEC11 and binding with the n2 domain and the R^20^R^21^ motif, which does not affect K^+^ channel binding (Grefen et al., 2010a) but may be important for channel binding exchange with SEC11. These findings are discussed further below.

## DISCUSSION

There is now a substantial body of evidence indicating that the Qa-SNARE SYP121 and its homologs in other species coordinate the rates of secretory vesicle traffic and solute uptake to accelerate cell expansion (Geelen et al., 2002; Honsbein et al., 2009; Grefen et al., 2010a, 2015; Karnik et al., 2013b, 2015). These studies have shown that solute uptake, especially of K^+^, is promoted by K^+^ channel binding with the Qa-SNARE and, in complementary fashion, that secretory traffic is promoted by SYP121 binding with the channels. Notably, SYP121 binds to the voltage sensor of the channels, commandeering this domain to confer a voltage dependence on secretory traffic and couple the rate of vesicle fusion to the overall transport activity at the plasma membrane (Grefen et al., 2015). Assembly of SYP121 within the SNARE complex, and the final stages of vesicle fusion, are regulated by the SM protein SEC11, which acts both to hold the Qa-SNARE in a silent, fusion-incompetent form and to stabilize the final SNARE assembly during fusion (Karnik et al., 2013b, 2015). Like other SM proteins, binding of SEC11 to SYP121 depends on residue F^9^ near the N terminus of SYP121 and thus overlaps with the K^+^ channel-binding motif F^9^xRF of the Qa-SNARE.

Although it might be anticipated that SEC11 and the K^+^ channels compete for SYP121 binding at its N terminus, such evidence has proven difficult to come by. Furthermore, in vitro pulldown assays have shown that binding of the full-length SEC11 is only partly reduced when paired with the SYP121^F9A^ mutant. In this context, it is important to note that binding can be localized to the N-terminal 39 residues, and the corresponding binding domain of the SM protein, SEC11^Δ149^, is sufficient to block secretory traffic with the native SYP121 (Karnik et al., 2013b, 2015). These results suggested the possibility of an additional domain contributing to SEC11 binding within the N-terminal region of SYP121. Our discovery of the residue pair R^20^R^21^ of SYP121 as an additional motif for binding with SEC11 now resolves this puzzle. We report (1) that this unique residue pair is critical for the selective binding of the SM protein; (2) eliminating this site alone blocks SYP121-mediated traffic, much as does the expression of SEC11^Δ149^, and in a manner that is not additive with SEC11^Δ149^ block; and finally (3) we show that mutation of the R^20^R^21^ residue pair, unlike the SYP121^F9A^ mutation, retains a substantial element of the link between vesicle traffic and osmotic solute uptake, blocking traffic without uncoupling solute and K^+^ uptake. These findings distinguish functionally the R^20^R^21^ SEC11-binding motif from that of the F^9^xRF motif for binding of both SEC11 and the K^+^ channels, and they suggest a mechanism by which the SEC11 binding exchange may facilitate the transition to SNARE complex assembly together with the channels.

### The R^20^R^21^ Pair Defines a Binding Motif Unique to SYP121

SEC11 was first identified as an interactor with the Qa-SNARE SYP111 during cell division (Waizenegger et al., 2000; Assaad et al., 2001) and, like SYP121, its interaction with SYP111 also depends on the N-terminal region of the Qa-SNARE (Park et al., 2012). Alignment of SYP111, SYP121, and its closest homolog SYP122 reveals high-level sequence similarity among these three SNAREs (Supplemental Fig. S3), notably the conserved F^9^ residue. From sequence alignment of SYP121 with SYP122, which does not bind SEC11 as strongly, we identified three regions (Fig. 3) within the unstructured N terminus of 39 amino acid residues. One of these regions, designated n1, overlaps with the F^9^xRF motif associated with K^+^ channel binding. Surprisingly, in addition to this region, a second region of eight residues within SYP121, n2, also suppressed SEC11 interaction when exchanged with the corresponding sequence from SYP122. A detailed analysis by Ala-scanning mutagenesis uncovered two key Arg residues, R^20^ and R^21^, displaced some 11 residues from the conserved F^9^, that strongly suppressed SEC11 interaction with SYP121 both in yeast (Fig. 3) and in vivo when assayed by FRET (Fig. 4).

The R^20^R^21^ motif is unique to SYP121 (Supplemental Fig. S3), suggesting a specialization in the interactions engaged by its binding with SEC11. This motif also separates SYP121 binding with SEC11 from that of the K^+^ channels (Grefen et al., 2010a; Honsbein et al., 2011). Previous studies led to the proposal of a handshake, or binding-exchange model, for SEC11 interactions in regulating SNARE complex assembly with SYP121 and its cognate partners, notably VAMP721 (Karnik et al., 2013b). This model accounted for SYP121 binding with the K^+^ channels, which is important for accelerating secretory traffic (Grefen et al., 2015). It also addressed the opposing effects on channel gating of SYP121 and VAMP721 binding with the K^+^ channel (Honsbein et al., 2009; Zhang et al., 2015, 2017) and their complementary binding with SEC11 (Karnik et al., 2013b). Thus, the handshake model proposed a binding exchange between SEC11 and the K^+^ channels as a key step driving the transit of SEC11 between its two binding modes, one with the closed, fusion-incompetent Qa-SNARE and the second with the SNARE complex assembly. In effect, a competition of the K^+^ channels with SEC11 for binding with SYP121 was seen to facilitate a transitory release of the Qa-SNARE from SEC11, thereby allowing SYP121 assembly with its cognate partners. Our findings here refine this scenario, with channel binding displacing SEC11-SYP121 binding to an intermediate conformation associated with the R^20^R^21^ motif prior to SNARE complex assembly.

In this context, it is noteworthy that SEC11 and SYP121 are expressed constitutively through the plant while the other SEC11 partner, SYP111, is found only in the cell plate during cytokinesis (Lauber et al., 1997). The embryo-lethal phenotype of the *sec11* mutation (Assaad et al., 2001) was originally associated with its impact on cell division, but a more recent and alternative interpretation draws on the importance of SEC11 for SYP121-dependent SNARE cycling and, as a consequence, the assembly of the cognate partners shared with other plasma membrane SNAREs (Karnik et al., 2013b, 2015). That SEC11 interactions build on a second, N-terminal binding motif on SYP121 underlines the importance of its coordination within the SNARE cycle with this Qa-SNARE. It follows that manipulations that tie up cognate partners needed in assemblies with other plasma membrane-related Qa-SNAREs are likely to have much broader impact than simply that of slowing traffic via SYP121.

### The SYP121 R^20^R^21^ Motif Separates the Actions of SEC11 and the K^+^ Channels on Secretory Traffic

Like other SM proteins, binding to the SYP121 N terminus relies on a minor cleft on the surface of SEC11 that is thought to coordinate binding and transitions of the Qa-SNARE between the closed, fusion-incompetent conformation and the open, fusion-competent conformation (Karnik et al., 2017). The finding that SEC11 binding relies also on a second site within the SYP121 N terminus is consistent with earlier pulldown data showing that the SYP121^F9A^ mutant retains significant binding capacity for SEC11, but it does not rule out interactions associated with its major cleft. To address this question, we examined SYP121 binding with the SEC11^Δ149^ peptide, which incorporates the minor SM cleft but lacks the structures forming the major cleft (Karnik et al., 2013b). These experiments (Fig. 5) were unambiguous, demonstrating that the SYP121^R20AR21A^ mutant eliminated binding with the SEC11^Δ149^ peptide.

SEC11^Δ149^ on its own also suppresses secretory traffic. Because traffic through both SYP121- and SYP122-dependent pathways was affected, but was specifically dependent on the presence of the native SYP121, Karnik et al. (2015) concluded that the block must arise through a sequestration of the cognate SNARE partners. Thus, we expected that SEC11^Δ149^ should block traffic in plants expressing each of the SYP121 constructs, including the SYP121^R20AR21A^ mutation. We did not anticipate that the SYP121^R20AR21A^ mutation on its own would block traffic. Furthermore, traffic block by the SYP121^R20AR21A^ mutant was not additive with that of SEC11^Δ149^ (Figs. 6 and 7). The simplest explanation for these findings is that the SYP121^R20AR21A^ mutant mimics a conformational state analogous to that of the SEC11^Δ149^-bound Qa-SNARE. The effects thus provide in vivo validation of the binding studies in yeast (Fig. 5).

Finally, to assess the functional effects of the R^20^R^21^ motif in the plant, we generated Arabidopsis lines expressing the key constructs, including SYP121^R20AR21A^, in the *syp121* background. We found that eliminating SYP121 promoted hypocotyl and root growth (Fig. 8), much as previous reports had shown that *syp121* mutant plants have larger epidermal cells, although no difference in rosette area was seen when compared with the wild type (Karnik et al., 2015). These findings suggest a moderating effect of SEC11- and SYP121-dependent growth that, in its absence, promotes early gains through parallel trafficking pathways, but without long-term benefits.

Analysis of hypocotyl growth, root growth, and K^+^ and total osmotic contents also showed a clear distinction between mutations associated with the F^9^xRF and the R^20^R^21^ motifs. Here, the SYP121n1 mutation showed accelerated root and hypocotyl growth and decreases in both K^+^ and total osmotic contents, whereas the SYP121n2 and SYP121^R20AR21A^ mutants showed accelerated growth but with only modest changes in K^+^ content and no effect on total osmotic content. Mutations affecting the F^9^xRF motif and K^+^ channel binding were shown previously to uncouple vesicle traffic from K^+^ and solute uptake (Honsbein et al., 2009; Grefen et al., 2010a, 2015). By contrast, mutations associated with the R^20^R^21^ motif showed functional differences, notably an intermediate level of K^+^ contents in the SYP121^R20AR21A^ mutant. These findings suggest a primary impact through the interaction with SEC11, rather than with the channels, and thus indicate a second site for SEC11 binding independent of the shared F^9^xRF motif.

**Figure 9.**
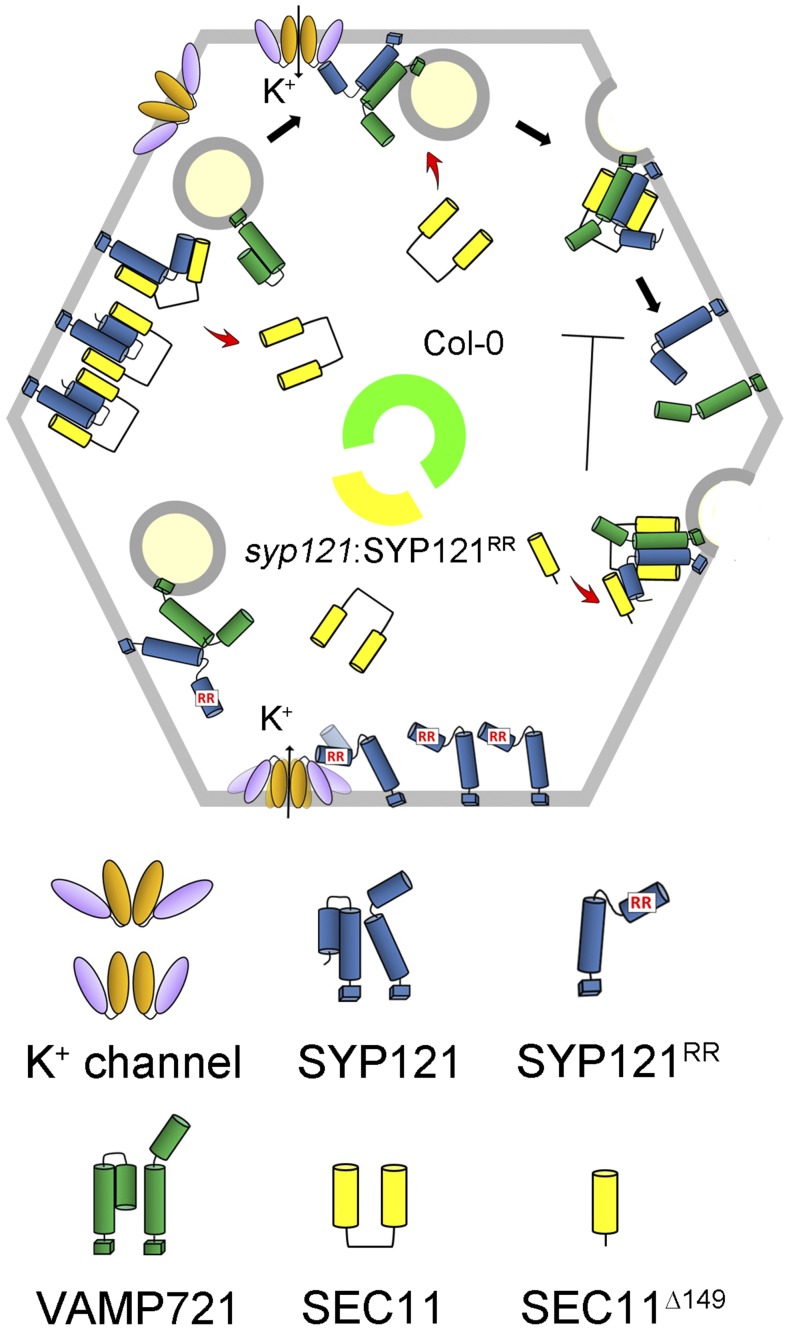
Proposed model for SYP121-related secretory traffic, the interaction between SYP121 and SEC11, and their uncoupling by the SYP121^R20AR21A^ mutant. Wild-type plants (Columbia-0 [Col-0], green arc) utilize SEC11 in facilitating SYP121 activation for interaction with VAMP721 and the K^+^ channels, stabilizing the SNARE complex for vesicle fusion, and the SEC11^Δ149^ fragment competes to prevent SNARE complex disassembly (Honsbein et al., 2009; Karnik et al., 2013b, 2015; Grefen et al., 2015; Zhang et al., 2015). In the *syp121* mutant complemented with SYP121^R20AR21A^, SEC11 binding for SNARE complex assembly with SYP121 is blocked, although K^+^ channel activation by the Qa-SNARE is retained, thereby suppressing SYP121-associated traffic but not K^+^ channel activity.

How might the R^20^R^21^ motif in SYP121 affect the SEC11 binding? One plausible sequence of events is shown in Figure 9 and incorporates knowledge from these several previous studies. In the wild-type plant (Fig. 9, central green arc), SYP121 in its closed conformation is stabilized by N-terminal binding with SEC11. During the membrane fusion cycle, K^+^ channel binding helps to recruit SYP121 and VAMP721 and releases SEC11; channel binding also promotes channel gating and K^+^ uptake. With SNARE complex assembly, SEC11 binding via its major cleft stabilizes the complex and after membrane fusion facilitates SNARE release. SEC11^Δ149^ binds with the N terminus of SYP121 and promotes an intermediate conformation that prevents the release of SNARE and thereby blocks traffic. The SYP121^R20AR21A^ mutation (Fig. 9, central yellow arc) introduces a similar, intermediate conformation to the Qa-SNARE, even without SEC11. The effect in this case is to suppress the SNARE cycle while still retaining partial K^+^ channel binding. Clearly, this is only one of several possible interpretations, but regardless of the detail, all interpretations must now accommodate the functional separation between binding SEC11 motifs on SYP121.

In conclusion, we identify two SEC11-binding motifs within the N terminus of SYP121. One motif is centered on residues F^9^xRF, which are also important for K^+^ channel binding. The second, previously unrecognized motif is associated with residues R^20^R^21^ and is specific for SEC11 interaction with SYP121. The R^20^R^21^ motif appears to confer SEC11 selectivity in binding with SYP121, and this interaction suggests a unique binding exchange of the Qa-SNARE with SEC11 during SNARE complex assembly, with consequences for plant growth and K^+^ and solute accumulation.

## MATERIALS AND METHODS

### Molecular Biology

Entry clones of SYP121, SYP122, SYP122^ΔC^, the SYP121 chimeras, iLOV, SEC11, and SEC11^Δ149^ in pDONR207 were described previously (Grefen et al., 2010a; Grefen and Blatt, 2012; Karnik et al., 2015) and used in Gateway reactions with pDONR221-P1P4 or pDONR221-P3P2 to generate 2in1 destination constructs (Grefen and Blatt, 2012). The site mutants were generated by site-directed mutagenesis with primers designed by SDM-Assist software (Karnik et al., 2013a) to include unique silent restriction sites along with the desired mutation for later identification via by restriction endonuclease digestion. Gateway destination clones were generated using LR Clonase II (Life Technologies). Gateway entry clones and destination clones were amplified using Top10 cells (Life Technologies) with the appropriate antibiotic, either 20 mg L^−1^ gentamicin or kanamycin for entry clones or 100 mg L^−1^ spectinomycin for destination clones.

For SUS assays, SEC11 was recombined in vector pMetOYC-Dest (Karnik et al., 2015) and SEC11^Δ149^ peptide was recombined in vector pEXG2Met-Dest as bait (Zhang et al., 2018). The SNARE constructs were recombined in vector pNX35-Dest (Grefen et al., 2009) as prey. For FRET analysis, the vector pFRETcg-2in1-CC (Hecker et al., 2015) was used to generate C-terminal mCherry-tagged SNARE constructs and C-terminal GFP-tagged SEC11. For secretion analysis and phenotype analysis, SYP121 and SYP121 chimeras were cloned in pUB-Bic-Dest (Grefen et al., 2010b) under the control of the UBQ10 promoter. The traffic rescue vector pTecG-2in1-CC with or without SEC11^Δ149^ was described previously (Karnik et al., 2015).

### mbSUS Assays

mbSUS assays were performed as described before (Grefen et al., 2009, 2010a). Bait constructs were transformed into yeast strain THY.AP4, and prey constructs were transformed into THY.AP5. Ten to 15 yeast colonies were selected and inoculated into selective media (CSM_-LM_ for THY.AP4 and CSM_-MTU_ for THY.AP5) for overnight growth at 180 rpm and 28°C. Liquid cultures were harvested and resuspended in yeast peptone dextrose (YPD) medium. Yeast mating was performed in sterile PCR tubes by mixing equal aliquots of yeast containing bait and prey constructs. Aliquots of 5 µL were dropped on YPD plates and incubated at 28°C overnight, and 1-mL aliquots were pelleted, ground in a ball mill, and frozen in liquid N_2_ for later immunoblot analysis (Karnik et al., 2013b). Colonies were transferred from YPD onto CSM_-LMTU_ plates and incubated at 28°C for 2 to 3 d. Diploid colonies were selected and inoculated in liquid CSM_-LMTU_ medium and grown at 180 rpm and 28°C overnight before harvesting by centrifugation and resuspension in sterile water. Serial dilutions at OD_600_ = 1 and 0.1 in water were dropped (5 µL per spot) on CSM_-AHLMTU_ plates with added Met. Plates were incubated at 28°C, and images were taken after 3 d. Yeast was also dropped on CSM_-LMTU_ control plates to confirm mating efficiency and cell density, and growth was imaged after 24 h at 28°C. To verify expression, yeast was harvested and extracted for protein immunoblot analysis using commercial HA antibody for prey and commercial VP16 antibody (Abcam) for the bait.

### Plant Transformation and Growth Analysis

Seedlings of Arabidopsis (*Arabidopsis thaliana*) wild type (Columbia-0) as well as *syp121-1* (*syp121*) and *syp122* mutants (Honsbein et al., 2009) were transformed transiently with *Agrobacterium tumefaciens* GV3101 as described before (Grefen et al., 2010b). The stable Arabidopsis lines were generated by floral dip (Clough and Bent, 1998), and transformed seeds were selected by growth on 50 mg L^−1^ phosphinothricin (Basta; Bayer Cropscience). Homozygous T4 lines were verified for expression of the transgene by immunoblot analysis, and at least two independently transformed lines were chosen for experiments.

For root phenotypic analysis, plants were grown on vertical 0.7% (w/v) agar plates and in liquid culture with defined minimal salts medium including 1 mm K^+^ (Zhang et al., 2015). Plants were germinated and grown at 22°C and 100 mmol m^−2^ s^−1^ photosynthetically active radiation of continuous light for 10 d. The root lengths were determined in three independent experiments from more than 10 seedlings per line using EZ-Rhizo (Armengaud et al., 2009). The same group of plants was harvested, ground in liquid nitrogen, and resuspended in sterilized water. K^+^ contents were determined by the rapid freeze-thaw method (Chen et al., 2016). Whole seedlings (20–30 plants) were collected in 1.5-mL tubes and sealed with Parafilm. Tubes containing leaf samples were frozen in −80°C and thawed at room temperature, with the freeze-thaw cycle repeated four times. Samples were then centrifuged, 10 µL of supernatant from each sample was diluted in distilled water, and K^+^ was determined by flame photometry (Sherwood 410). Ten microliters of the same supernatant was used to determine the osmolality using a vapor pressure osmometer (Vapro 5520; Wescor).

For hypocotyl elongation analysis, seeds were sterilized and placed on 0.5× Murashige and Skoog medium containing 0.8% (w/v) agar and 0.5% (w/v) Suc. After 3 d of stratification at 4°C, plates were placed under a red filter for 3 h to promote germination and left vertically in the dark for 6 d. Plates were scanned and hypocotyl lengths measured using ImageJ v.1.52 (http://rsb.info.nih.gov/ij/). No statistically significant differences were found between lines with the same construct; these data were therefore pooled.

Wild-type tobacco (*Nicotiana tabacum*) plants were grown in soil at 26°C and 70% relative humidity on a 16/8-h (day/night) cycle for 4 to 6 weeks. Plants with three to four fully expanded leaves were selected for transformation (Blatt and Grefen, 2014).

### Confocal Microscopy

Arabidopsis seedlings were imaged after 3 d of cocultivation with *A. tumefaciens* to transform the root epidermis with vector constructs with the gene fusions of interest (Blatt and Grefen, 2014). Tobacco leaves were infiltrated with *A. tumefaciens* GV3101 as described before (Tyrrell et al., 2007). Confocal images were collected using a Leica TCS SP8-SMD confocal microscope and a 20×/0.75NA objective lens. For FRET studies, the GFP fluorescence was excited with 488 nm and collected over 500 to 535 nm; the FRET signal was collected over 590 to 645 nm. mCherry fluorescence was also detected separately with excitation at 552 nm. Changes in FRET were calculated as the FRET-mCherry fluorescence ratio and normalized to the GFP signal to correct for expression. Bleedthrough from GFP fluorescence over 590 to 645 nm and from mCherry absorption at 488 nm was corrected based on the respective fluorophore signals and their absorption and emission spectra. For SYP121^R20A,R21A^ localization, the GFP fusion was analyzed by exciting with 488-nm light, and fluorescence was collected over 500 to 535 nm. For secYFP trafficking studies, the GFP fluorescence was excited at 458 nm and collected over 500 to 535 nm. The YFP fluorescence was excited at 514 nm and collected over 535 to 590 nm. Traffic block was determined from the YFP-GFP fluorescence ratio. All fluorescence images were rendered as 3D projections, and fluorescence intensities were quantified from the roots after subtracting the mean background fluorescence collected from the untransformed seedlings grown in parallel.

### Plant Extraction and Immunoblot Analysis

Leaf tissue was harvested, weighed, flash frozen, and ground in liquid N_2_. Yeast aliquots were obtained as indicated above. Equal weights of material were sonicated and suspended 1:1 (w/v) in homogenization buffer that contained 500 mm Suc, 10% (v/v) glycerol, 20 mm EDTA, 20 mm EGTA, 1× Protease Inhibitor (Roche), 10 mm ascorbic acid, 5 mm DTT, and 50 mm Tris-HCl, pH 7.4. Samples were centrifuged at 13,000*g* for 10 min at 4°C, supernatants were diluted 1:1 in loading buffer (Karnik et al., 2013b), and proteins were separated by SDS-PAGE on 10% (v/v) acrylamide gels. Immunoblot analysis was carried out, after transfer to nitrocellulose filters and blocking, using primary commercial antibodies α-GFP and α-mCherry (1:5,000), α-HA (1:2,000), α-myc (1:5,000), and α-VP16 (1:10,000) and secondary goat anti-rabbit antibodies (Abcam). Detection was with the ECL Advance detection kit (GE Healthcare). All blots were recorded digitally and analyzed using ImageJ v. 1.48 (http://rsb.info.nih.gov/ij/).

### Statistical Analysis

Statistical analysis of experiments was reported as means ± se as appropriate, with significance determined by Student’s *t* test or ANOVA in SigmaPlot v.11.2 (Systat Software).

### Accession Numbers

Sequence data from this article can be found in the GenBank/EMBL data libraries under TAIR accession numbers At3g11820, At3g52400, At1g08560, and At1g12360.

### Supplemental Data

The following supplemental materials are available.

**Supplemental Figure S1.** The mutated Qa-SNARE SYP121^R20A,R21A^ localizes to the cell periphery.
**Supplemental Figure S2.** The expression of SYP121 chimeras and Ala substitution in the R^20^R^21^ motif in the *syp121* line.
**Supplemental Figure S3.** Alignment of the n1 and n2 segments of SYP111, SYP121, SYP122, SYP131, and SYP132.
